# Comparison of spatial and non-verbal reasoning abilities in veterinarians in the fields of radiology and surgery

**DOI:** 10.3389/fvets.2024.1438062

**Published:** 2024-11-01

**Authors:** Juan Claudio Gutierrez, Steven D. Holladay

**Affiliations:** ^1^School of Veterinary Medicine, University of California, Davis, Davis, CA, United States; ^2^College of Veterinary Medicine, University of Georgia, Athens, GA, United States

**Keywords:** spatial ability, anatomy, visual reasoning, veterinary radiologists, veterinary surgeons

## Abstract

Spatial ability tests measure capacity for mentally understanding and interpreting three-dimensional images. Such skills have been found to be predictive for anatomical learning success and proficiency in human and veterinary medical students. Veterinarians in the radiology and surgery field develop high levels of three-dimensional topographic anatomic understanding through exposure to anatomy portions of the veterinary curriculum, followed by highly specialized residency programs. Validated testing tools were used to compare spatial and general non-verbal reasoning abilities in veterinarians in the field of radiology (radiology group, RG) and veterinarians in the field of surgery (surgery group, SG). These tests were: Guay’s Visualization of Views Test: Adapted Version (GVVT), the Mental Rotation Test (MRT), and Raven’s Advanced Progressive Matrices Test, short form (APMT). Results showed a significant difference for GVVT scores in favor of the RG (15.2 ± 0.3 and 12.3 ± 0.4, respectively, *p* < 0.05). There were no significant differences in scores for MRT and APMT between the RG and SG. There was a significant positive correlation between spatial ability tests scores and general non-verbal reasoning test scores for the RG but not for the SG. Future studies are planned to determine if the RG innately possess high spatial and reasoning skills, and to expand the present findings to other veterinary specialty areas.

## Introduction

Spatial visualization ability is defined as the ability to mentally rotate two and three-dimensional figures. This cognitive process is involved in situations in which mental representations of objects are formed based on two-dimensional or other visual displays. This is of relevance especially to a wide spectrum of professional disciplines, including engineering, architecture, mathematics, computer sciences, natural sciences and a variety of medical disciplines particularly radiology and surgery, and relates to the process by which internal three-dimensional representations of objects are mentally generated based on the assimilation and integration of a series of two-dimensional spatial displays. In the case of Stanford University, new dentistry students are required to take spatial and non-verbal reasoning tests as part of the admission process into the program (1). This is done through use of standardized tests that have been developed to quantify spatial and non-verbal reasoning abilities. Guay’s Visualization of Views Test (GVVT) and the Mental Rotation Test (MRT) are two such tests commonly used to assess spatial ability. General non-verbal reasoning ability is another skill that positively correlates with scores on GVVT and MRT and can be measured by standardized tests (2–4). Non-verbal reasoning tests employ a series of perceptual analytical reasoning problems, with each often in the form of a matrix (5). Raven’s advanced progressive matrices test, short form, (APMT) is one such matrix-based test used to measure non-verbal reasoning ability (6, 7).

Human medical students were found to possess higher spatial ability than students enrolled in other science disciplines, and to show greater spatial ability improvement with progression through the medical curriculum (8). Related studies in veterinary medical students found increases in spatial ability scores at both 32 and 64 weeks into the DVM curriculum as compared to student entry levels (7). These authors suggested the experiencing of substantial anatomic dissection, an intense, highly visual and 3-dimensional portion of the curriculum, may play an important role in the increase of spatial skills. At the undergraduate level, Guillot et al. similarly found significant positive correlation between spatial ability and academic outcome in anatomy courses. These authors also detected increasing spatial ability as undergraduate students progressed through curricula that included anatomy courses (9). Based on such collective observations, Lufler et al. (10) hypothesized that early tutor availability or similar interventions may be useful for students who enter medical programs of study with lower spatial ability scores in the United States. Such intervention would require spatial ability testing of incoming medical students, which is not now a general practice of medical colleges.

The present report utilized three standard tests designed to measure spatial and non-verbal reasoning abilities (GVVT, MRT, and AMPT). These were taken on a volunteer basis by veterinary radiologists and veterinary surgeons. Radiologists and surgeons were selected for this initial study because both specialties require prolonged training that included considerable anatomy. Radiologists and surgeons were also felt to be somewhat differentially trained anatomically, as imaging may require more whole-body anatomic knowledge (head-to-toe; all body cavities) while surgeons may anatomically be more region/structure-focused (soft-tissue surgeons; orthopedic surgeons). The hypotheses being tested were: (1) There is no significant difference in visual–spatial and general non-verbal reasoning abilities between veterinary radiologists and surgeons as measured by three standardized tests. (2) There is a positive correlation between spatial ability and general non-verbal reasoning ability in both veterinary radiologist and surgeons.

## Materials and methods

Previously validated testing tools were used to measure spatial and general non-verbal reasoning abilities in veterinarians in the fields of radiology and surgery. Participating veterinary radiologists and surgeons were not at any specific points in their careers, and included trainees as well as individuals who have completed residencies but were not yet board certified. The tests required 34 total minutes to complete and were delivered in an online platform provided by Stanford University, Division of Clinical Anatomy. Stanford University has selected and established the use of these tests with medical and dentistry students.

Participation in this preliminary experiment was voluntary. Radiologists and surgeons were recruited through communication with the American College of Veterinary Radiology (ACVR) and the American College of Veterinary Surgeons (ACVS), respectively. The study was considered exempt from formal review by the University of California Institutional Review Board.

Two groups of participants were enrolled for testing:

1. Veterinarians in the surgery field (Surgery group, SG). These included trainees, certified and non-certified veterinarians (*N* = 23).
2. Veterinarians in the radiology field (Radiology group, RG). These included trainees, certified and non-certified veterinarians (*N* = 53).

Three tests were taken by all participants:

1. Guay’s Visualization of Views Test: Adapted Version (GVVT): this test measures spatial ability. The test measures the ability to correctly recognize 3-D objects viewed from different positions. It includes 24 questions, is timed at 8 min to complete, and is a modified and validated version of The Purdue Visualization of Views Test. Questions on this test show rotated images of 3-D objects suspended in a transparent cube.

Individuals being tested must identify the correct corner of the cube from which a virtual picture of the suspended object was taken. The picture of the suspended object is shown above the cube in each question. Incorrect answers incur a penalty of 1/6 of a point, making the possible range of scores −4 to 24 (4, 5).

2. Mental Rotations Test (MRT): this is a second test of spatial ability. The test requires selection of 3-D objects that are identical in shape to a reference object, but shown in different rotational orientations. This test was first used by Vandenberg and Kuse (11). This test measures ability to mentally rotate complex 3-D shapes in order to find a match. The 40-item test is administered in two 20-item parts timed at 3 min each, for a total of 6 min. Participants receive 1 point for each correct answer and −1 point for each incorrect answer, giving a range of possible scores from −40 to 40.

3. Raven’s Advanced Progressive Matrices Test, short form (APMT): this test measures non-verbal general non-verbal reasoning ability (visual reasoning). This 12-question, 12-min test is a sub-set of the original full-length Raven’s Advanced Progressive Matrices Test, which was validated by Bors and Stokes (2). The test requires correct identification of the missing pattern in a complex design of patterns or diagrams, from a set of 8 choices. Individuals tested are not penalized for incorrect answers, such that scores fall between 0 and 12 (1, 3).

### Statistical analysis

Descriptive statistics and a Shapiro–Wilk test were performed to evaluate the presence or absence of normal distribution. A Mann–Whitney test for non-parametric data was used to compare test scores between the two groups. A Spearman’s correlation for non-parametric data was performed to determine if spatial ability test scores (GVVT and MRT) were correlated to non-verbal reasoning test scores (APMT). The Spearman correlation ranges from −1 to 1 with 0 indicating there is no tendency for the first variable to increase or decrease as the second variable increases. XLSTAT was used to perform the data analysis. For all analyses a *p* < 0.05 was considered significant.

## Results

A total of 23 veterinarians in the surgery field, and 52 veterinarians in the radiology field took all 3 online tests. Results from the Mann–Whitney test showed that mean scores on the GVVT were significantly higher in the RG (15.2 ± 0.3 for RG and 12.3 ± 0.4 for SG). Mean scores on the MRT and APMT did not show a significant difference between the RG and the SG, being 16.7 ± 0.4 and 14.4 ± 0.5, respectively for MRT and, 7.3 ± 0.3 and 6.9 ± 0.5, respectively for APMT (Table 1).

**Table 1:** Mean ± SEM of surgeons’ and radiologists’ performance for the 3 tests. GVVT: Guay’s Visualization of Views Test, MRT: Mental Rotations Test, APMT: Raven’s Advanced Progressive Matrices Test.

	GVVT	MRT	APMT
Test scores SG (*N* = 23)	12.3 ± 0.4	14.4 ± 0.5	6.9 ± 0.5
Test scores RG (*N* = 52)	15.2 ± 0.3*	16.7 ± 0.4	7.3 ± 0.3

*p < 0.05.

When performing a Spearman’s correlation within groups between the spatial ability scores (GVVT and MRT) and the non-verbal reasoning ability scores (APMT), a positive correlation was found between the GVVT scores and the APMT scores and, a stronger positive correlation was found between the MRT scores and the AMPT scores for the RG. The same comparison of scores yielded no significant differences in the SG (Table 2).

**Table 2:** Spearman’s correlation for GVVT (Guay’s Visualization of Views Test) and MRT (Mental Rotation Test) scores vs. APMT (Raven’s Advanced Progressive Matrices Test) scores within groups.

	GVVT/APMT	MRT/APMT
SG (*N* = 23)	−0.11	+0.21
RG (*N* = 52)	+0.33*	+0.55**

*p < 0.05, **p < 0.0001.

## Discussion

Due to the three-dimensionality of the field of anatomy, it has been suggested that anatomic proficiency may positively correlate with spatial ability (6, 7). Studies by Lufler et al. (10) found that medical students experienced significant visual spatial improvements during participation in the medical gross anatomy courses in the medical program. Similar benefits have been suggested for dentistry students (12). Studies by Gutierrez et al. (7, 13) established that spatial and non-verbal reasoning abilities also improved in 1st-year veterinary medical students exposed to cadaver dissection labs in an integrated curriculum. Provo et al. (14) found a significant correlation between spatial ability scores and performance on anatomy examinations. These authors therefore suggested that students with low spatial ability are at increased risk of poorer academic outcome in anatomy. Veterinarians in the fields of radiology and surgery have a substantial knowledge of topographic anatomy due to their extensive training and the anatomic demands of their disciplines. For the same reason veterinarians of both disciplines might be expected to possess similar levels of spatial abilities when tested by standardized tests. Indeed, it has been suggested that based on review of the existing cognitive psychological literature and based on the assumption that spatial ability is of increasing and critical importance to high-level performance of clinical radiologists, it has been proposed that consideration should be given to the testing of visuospatial ability as part of the selection process for prospective applicants to radiology training programs (15).

For surgeons, it has in like manner been suggested that visual–spatial ability is related to competency and quality of results in complex surgery, and could potentially be used in resident selection, career counseling, and training (16). Interestingly, studies by Keehner et al. (17) found that university students with high performance in spatial and visual reasoning tests were more rapid learners of surgical laparoscopic techniques by virtual reality than those with lower performance. No previous information has been published directly comparing spatial abilities in veterinarians in the fields of radiology and surgery, who share highly visible disciplines but also differ in many ways. In the present testing, the RG scored significantly higher than the SG on one of the three tests, the GVVT. Unlike the SG, the RG also showed a significant positive correlation between two of the spatial ability tests, the GVVT and the MRT, and the general non-verbal reasoning ability APMT test. Such positive correlations are not unusual, making it possibly more noteworthy that the SG did not display the same correlations (6).

The GVVT and MRT are again tests of spatial abilities, designed to determine ability to mentally recognize and manipulate 3-D objects. Raven’s APMT test is related in quantifying general non-verbal reasoning ability, through ability to identify missing patterns in complex designs. Higher GVVT scores among radiologists may be consistent with imaging analyses the radiologists perform through much of their workday, as compared to lesser time surgeons may spend in actual surgeries. In addition to standard radiographs, radiologists also spend considerable time in cross-sectional based imaging (ultrasound, CT, MRI) where images may be imported into software programs that allow image rotation in desired directions as well as passing of planes through images to sequentially visualize surfaces of areas of interest. Correctly interpreting radiological images is in these ways highly dependent on visual spatial ability as well as the platform anatomic knowledge mentally projected into each interpreted image. A role in the need for spatial visualization skills in such interpretation was supported by Rengier et al. (18), who found that use of interactive 3-D imaging software in medical students improved radiology education imaging diagnostic skills and visual–spatial ability. The students tested by these investigators were 4th and 5th-year medical students taught by experienced radiologists, who were tested immediately before and after their radiology course. Spatial ability scores in these students were improved by 11.3% at the end of the radiology course, similar to observations made by the present authors in 1st-year veterinary students (18). Such results could be consistent with the complex imaging analysis that radiologists must perform in their everyday work. In fact, one of the key factors for correctly interpreting radiological images and successfully learning anatomy is visual spatial ability. It has been suggested that the integration of interactive 3D image post-processing software into undergraduate radiology education effectively improves radiological reasoning, diagnostic skills, and confidence as well as visual–spatial ability. Consequently, medical students felt better prepared for every day clinical practice (15).

A major limitation of the present study may be the small number of participants in the SG, and the lack of analysis of scores by sex. Spatial ability has shown to produce sex differences, in favor of males. Studies performed by Vorstenbosch et al. (8) revealed a significant difference in favor of male medical students in spatial ability tests. Studies by Gutierrez et al. (6), also reveal a difference in spatial ability scores in veterinary medical students in favor of males. In another study by the same authors, female spatial ability scores significantly improved when analyzed independently (male scores were not considered in the analysis) (13). In those studies, spatial and visual reasoning scores were obtained twice: before and after exposure to cadaver-dissection anatomy labs. Vandenberg and Kuse (11) revealed significant differences in MRT scores for undergraduate students: 19.06 for men and 13.17 for women. The latter studies expose a limitation of the present study due to the inability to analyze scores from males and females independently.

It will be important to repeat the present study with a larger number of participants, particularly veterinary surgeons. Despite efforts otherwise, a relatively small number of SG were surveyed (*N* = 23) among those working in this very busy discipline. The present analysis also did not attempt to further divide RG and SG groups by years of experience (in academia or different private sectors), board-eligible or specialty boarded, or sex. The present study did not attempt to examine difference by sex due to the small number of the SG participants available and because many participants chose to not answer the sex identifier question of the test. An interesting outcome of research of this sort can be the exploration of spatial ability tests utility as part of the admission processes for specialized training and residencies in veterinary radiology and surgery. Stanford University uses such standard tests in the admission process for Dentistry School.

## Conclusion

This preliminary study found: (1) significantly higher mean GVVT spatial abilities score in 53 RG as compared to 23 SG taking the test; (2) no significant difference in scores for MRT and APMT between RG and SG; and (3) a significant positive correlation between spatial ability tests scores and non-verbal reasoning test scores for the RG. Future studies should include higher numbers of participants, although such is challenging given these are both very busy specialty areas with relatively few members. This obstacle might be overcome in human as opposed to veterinary medical radiologists and surgeons, where greater member numbers are available. Future studies should also consider subgroups based on years of experience and sex. These studies might also expand to include additional highly-visual specialty areas including veterinary pathology and ophthalmology.

## Data Availability

The raw data supporting the conclusions of this article will be made available by the authors, without undue reservation.
